# Redefining the oceanic distribution of Atlantic salmon

**DOI:** 10.1038/s41598-021-91137-y

**Published:** 2021-06-10

**Authors:** Audun H. Rikardsen, David Righton, John Fredrik Strøm, Eva B. Thorstad, Patrick Gargan, Timothy Sheehan, Finn Økland, Cedar M. Chittenden, Richard D. Hedger, Tor F. Næsje, Mark Renkawitz, Johannes Sturlaugsson, Pablo Caballero, Henrik Baktoft, Jan G. Davidsen, Elina Halttunen, Serena Wright, Bengt Finstad, Kim Aarestrup

**Affiliations:** 1 Department of Arctic and Marine Biology, UiT The Arctic University of Norway, 9037 Tromsø, Norway; 2 Centre for Environment, Fisheries and Aquaculture Science, Lowestoft, UK; 3 Norwegian Institute for Nature Research, 9007 Tromsø/7034 Trondheim, Norway; 4 Inland Fisheries Ireland, 3044 Lake Drive, Citywest Business Campus, Dublin 24, Ireland; 5 NOAA Fisheries Service, Northeast Fisheries Science Center, Woods Hole, MA USA; 6 Laxfiskar, Mosfellsbaer, Iceland; 7 Servicio de Conservación de la Naturaleza de Pontevedra, 36071 Ponteverda, Spain; 8 National Institute of Aquatic Resources, Technical University of Denmark, Silkeborg, Denmark; 9 Department of Natural History, NTNU University Museum, 7491 Trondheim, Norway; 10 Department of Biology, NTNU University of Science and Technology, 7491 Trondheim, Norway

**Keywords:** Ecology, Ecology, Environmental sciences, Ocean sciences

## Abstract

Determining the mechanisms driving range-wide reductions in Atlantic salmon marine survival is hindered by an insufficient understanding of their oceanic ecology and distribution. We attached 204 pop-up satellite archival tags to post-spawned salmon when they migrated to the ocean from seven European areas and maiden North American salmon captured at sea at West Greenland. Individuals migrated further north and east than previously reported and displayed increased diving activity near oceanographic fronts, emphasizing the importance of these regions as feeding areas. The oceanic distribution differed among individuals and populations, but overlapped more between geographically proximate than distant populations. Dissimilarities in distribution likely contribute to variation in growth and survival within and among populations due to spatio-temporal differences in environmental conditions. Climate-induced changes in oceanographic conditions will alter the location of frontal areas and may have stock-specific effects on Atlantic salmon population dynamics, likely having the largest impacts on southern populations.

## Introduction

Temperatures have increased in the north Atlantic and Pacific oceans over the last few decades, causing large-scale oceanographic changes^1,2^ and northward distributional shifts of many species^3,4^. Diadromous Atlantic salmon (*Salmo salar*) and Pacific salmon (*Oncorhynchus* spp.) spawn and spend the juvenile phase in rivers and perform long-distance ocean feeding migrations^5,6^. It has been suggested that many of these species have expanded their marine feeding areas northwards^7,8^, resulting in longer migration distances to foraging areas.

In Europe and North America, the abundance of Atlantic salmon has generally declined since the 1970s^9,10^. One of the major hypotheses for the decline is reduced marine survival^9,10^. This has increased interest in the spatio-temporal ocean distribution of Atlantic salmon^11,12^ and the impacts of the ocean environment on individual growth and survival^13,14^. However, the causal links between ocean processes and survival remain elusive. A key element in identifying and evaluating factors that contribute to reduced survival is knowledge of migration routes, migration timing and feeding areas. With knowledge of the oceanic distribution of salmon from different regions, the causative mechanisms underlying the variation in growth and productivity can be better understood.

The Atlantic salmon is one of the world’s most studied fish, but detailed knowledge of its ocean distribution and behaviour is limited. Traditionally, information on the ocean migration originated from sampling and conventional tagging surveys based on mark and recapture methods^15,16^. More recently, genetic studies have disentangled novel aspects of the species’ ocean migration and distribution^17–19^. These previous studies have been spatially limited by primarily sampling from fisheries and surveys at the Faroes, Greenland and in the Norwegian Sea^20^ rather than over more widespread regions that Atlantic salmon are believed to use. They have also provided little information on detailed migration routes and behaviour.

The development of electronic tracking technologies has opened new possibilities to collect detailed spatially unbiased information on fish migrations and individual behaviour over large ocean areas^21,22^. For Atlantic salmon, the use of archival tags has expanded our knowledge of where they feed in the ocean^12,23–26^ and provided detailed data on individuals’ depth and temperature use^27–29^. However, archival tags have typically been used in studies of single populations, or in limited geographical areas, and included a low number of individuals.

To increase our understanding of the ocean distribution of Atlantic salmon, and to study variation in distribution between and within populations, we performed a large-scale study using pop-up satellite archival tags (PSATs) spanning populations from the southernmost to the northernmost part of the species’ distribution range. The study included post-spawned salmon from seven areas in Europe (from 42° N in Spain to 70° N in northern Norway) that were tagged as they were leaving the river and returning to the sea, and maiden North American salmon captured at sea at Western Greenland. The tags archived environmental data until detachment when they reported their location and transferred stored data to satellites. Our aims were to (1) map the horizontal ocean migration patterns of individual Atlantic salmon, (2) examine how their migration patterns were related to origin, travel distance, area use and diving frequency, and (3) quantify the overlap in feeding areas of salmon from the different regions.

## Results

**Figure 1:**
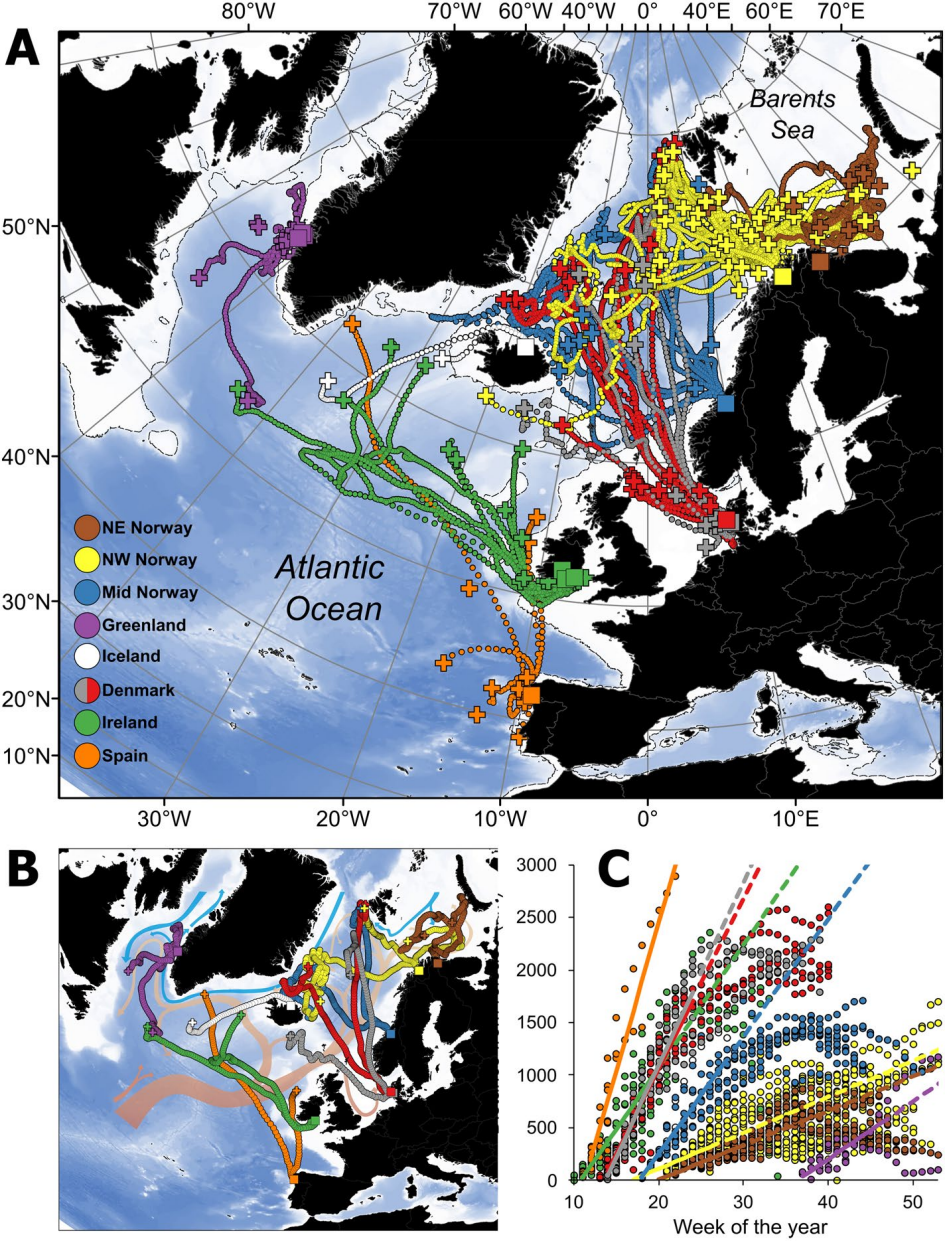
Migrations of Atlantic salmon tagged in eight different geographic areas. Release locations post-tagging are shown by squares (from 11 northeast Atlantic river catchments and at-sea at Western Greenland). (**A**) Estimated daily geographic location of 105 salmon (circles) from the release location. Crosses show the pop-up location of the tags of these salmon, as well as those for an additional 43 salmon for which detailed movement reconstructions were not possible. The dashed line shows the 500 m depth contour, while darker blue shading indicates increasing depth as per the GEBCO bathymetry^44^. (**B**) Example migrations of individual fish from each catchment and arrows showing main ocean current systems of warm (light brown arrows) and cold (blue arrows) water in the North Atlantic Ocean. (**C**) The weekly mean distance from the release location for salmon from each tagging group (circles, estimated as distance along a straight line from the release location). Fitted lines show the regression (significant for all groups) between week of the year and distance for the first 11 weeks after release only, corresponding to the time required to reach principal feeding areas for most populations (regression lines were extended as dashed lines past the first 11 weeks for display purposes only). Iceland was not included in (**C**) due to our small sample size of fish from this area. Maps were drawn using ESRI ArcGIS Desktop v10.5.

From the 204 tagged fish, data were obtained from the PSATs of 148 Atlantic salmon, of which 105 provided enough data for estimating complete migration paths while the remainder provided pop-up locations indicating the approximate position of the animals on the date the tag released. The migration tracks calculated for these fish showed that individuals migrated towards known oceanographic fronts, where branches of the North Atlantic current lie adjacent to cold polar waters, but the oceanic distribution differed among individuals and populations (Fig. 1). Most Norwegian and Danish salmon conducted a rapid northern or north-western migration in the North Atlantic Ocean (Fig. 1). However, salmon from northern Norway either migrated north-west in the northern Norwegian Sea toward Svalbard or the Greenland Sea, or north-east to the Barents Sea (Fig. 1). The northernmost recordings of Norwegian and Danish Atlantic salmon were from the west of Svalbard at latitudes of nearly 80° N. In contrast, Irish, Spanish and Icelandic salmon primarily migrated westward towards East Greenland (Fig. 1). Maiden North American salmon (genetically assigned) tagged at Western Greenland migrated south into the Labrador Sea during fall and winter.

**Figure 2:**
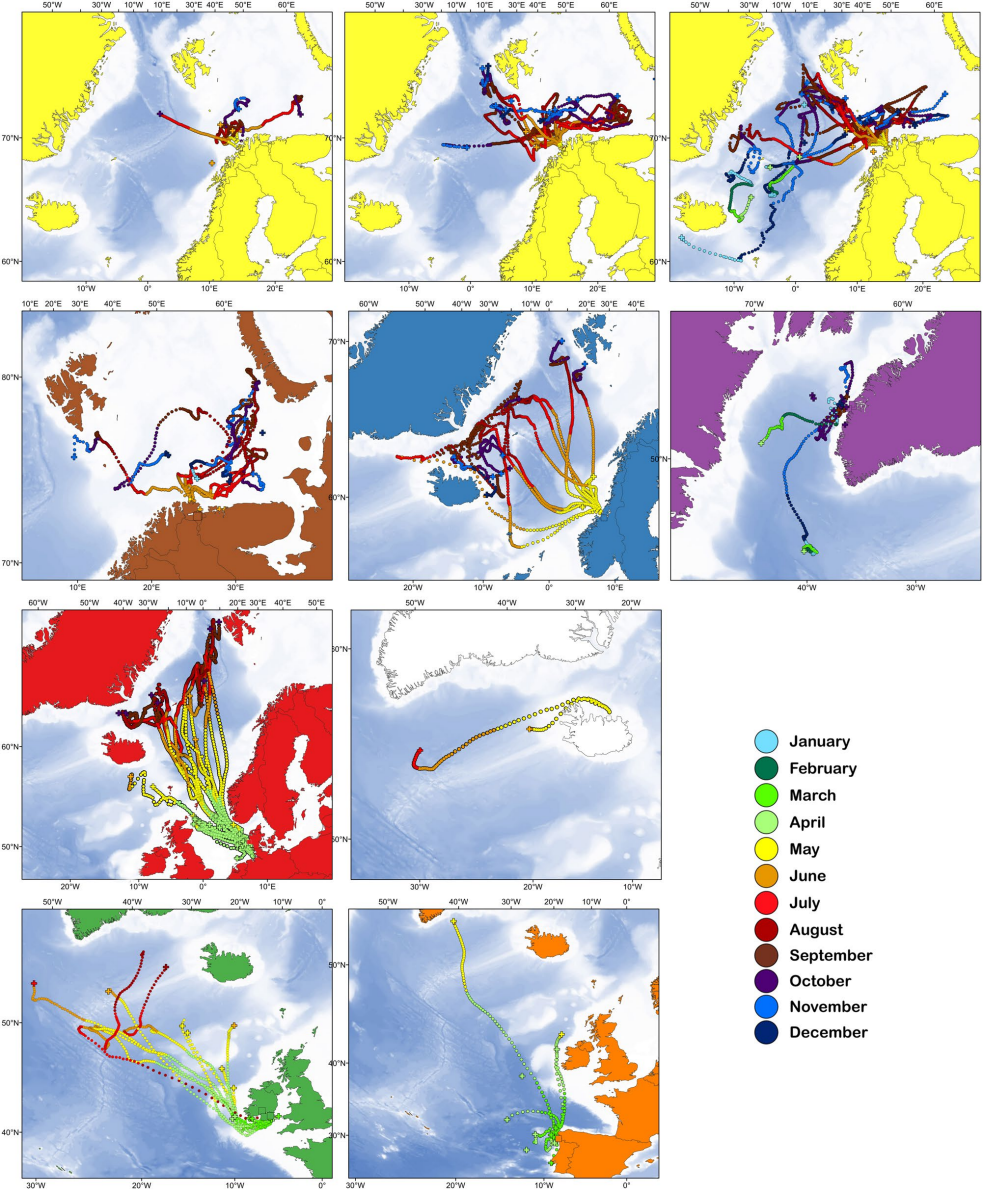
Migration tracks by month of individual Atlantic salmon tagged in seven geographic areas. Each panel shows the tracks of fish tagged in one geographic area (indicated by the color of the land masses in each map, corresponding to color codes in Fig. 1). Because of the large number of individuals tagged in the NW Norwegian population, the three uppermost panels show data from this population the three different years of tagging (2008–2010 left to right). Darker blue shading in the ocean indicates increasing depth as per the GEBCO bathymetry^44^. Annual data for the remaining population with multiple years of tagging is shown in Supplementary Figure S1. Maps were drawn using ESRI ArcGIS Desktop v10.5.

Most salmon rapidly migrated towards the oceanographic fronts (Fig. 1). Within each population, individuals commonly migrated in similar directions, although population-specific variation was noted (Fig. 1). Individuals covered distances of up to 2940 km from the release site, measured as a straight-line distance from the tagging site to the pop-up position (an individual from River Lerez in Spain with the longest migration), with fish released further south travelling the longest distances (Table 1; Fig. 1). For the populations with multiple years of tagging (i.e., salmon from 2 years in Denmark and 3 years in Northwest Norway, Table 1), the migration routes showed similar patterns between or among the years (Fig. 2).

**Table 1:** Overview of tagged post-spawned Atlantic salmon from eleven rivers in eight geographic areas in the north-east Atlantic and maiden salmon captured off Greenland. The number of fish tagged, tagging dates and fish body size is given for each river/location each year. The number of fish with tags providing data, the total number of days recording data, mean % of the data retrieved from the tags, mean distance migrated as a straight line from the river to the pop up location, and the maximum recorded swim depth of the fish are also given. *NW *north-western, *NE *north-eastern, *SD *standard deviation.

Location (area)	River	Year of tag-ging	Number of fish tagged	Dates of releasing tagged fish	Mean body length, cm (SD)	Mean body mass, kg (SD)	Number of fish with tags providing data	Number of days recorded	Mean % of data retrieved (SD)	Distance migrated, km, mean (SD, maximum)	Max recorded depth, m
NW Norway	Alta	2008	10	22 May	102 (7.2)	8.1 (1.6)	8	748	83 (34)	463 (232, 846)	459
		2009	20	29 May	98 (4.5)	7.0 (1.0)	17	2437	57 (29)	632 (297, 1108)	659
		2010	22	24–27 May	99 (5.6)	7.1 (1.0)	21	3752	49 (36)	686 (457, 2068)	634
NE Norway	Neiden	2009	10	31 May	96 (5.6)	6.2 (1.6)	10	1391	56 (24)	344 (241, 778)	397
		2010	7	30 May	83 (7.1)	4.0 (1.1)	5	334	48 (41)	144 (155, 348)	220
Mid Norway	Orkla	2010	20	5–6 May	98 (8.0)	6.7 (1.6)	10	1491	70 (18)	1039 (456, 1720)	644
Denmark	Skjern	2011	12	31 March	89 (8.2)	4.5 (1.4)	8	1186	55 (24)	1781 (846, 2603)	585
		2012	12	1 April	84 (3.7)	3.6 (0.5)	10	464	73 (18)	878 (618, 1988)	312
	Varde	2013	10	2 April	83 (3.6)	3.6 (0.7)	7	502	57 (40)	1396 (930, 2064)	468
		2014	10	3 April	82 (5.8)	3.3 (0.8)	7	414	66 (25)	997 (928, 2018)	247
Ireland	Blackwater, Suir	2010	17	11–25 March	74 (6.2)	3.0 (0.6)	10	502	90 (11)	796 (856, 2391)	870
	Suir, Nore, Barrow	2011	10	11–18 March	71 (5.2)	2.8 (0.6)	9	546	84 (23)	709 (717, 1978)	639
Iceland	Laxa	2010	6	4 May	79 (2.9)	2.9 (0.5)	2	99	95 (8)	940 (559, 1335)	505
Spain	Lérez	2013	7	14 March	74 (2.8)	2.8 (0.6)	6	550	87 (11)	963 (1104, 2971)	560
		2014	7	18 March	84 (6.3)	4.0 (1.1)	5	91	91 (10)	450 (483, 1277)	430
Greenland	NA (in sea)	2010	7	13–15 Sept	66 (2.3)	3.7 (0.5)	4	254	86 (23)	331 (327, 804)	768
		2011	17	14–27 Sept	66 (2.7)	3.5 (0.3)	9	312	93 (19)	279 (380, 1259)	672
Total			204				148		69	25,954

**Figure 3:**
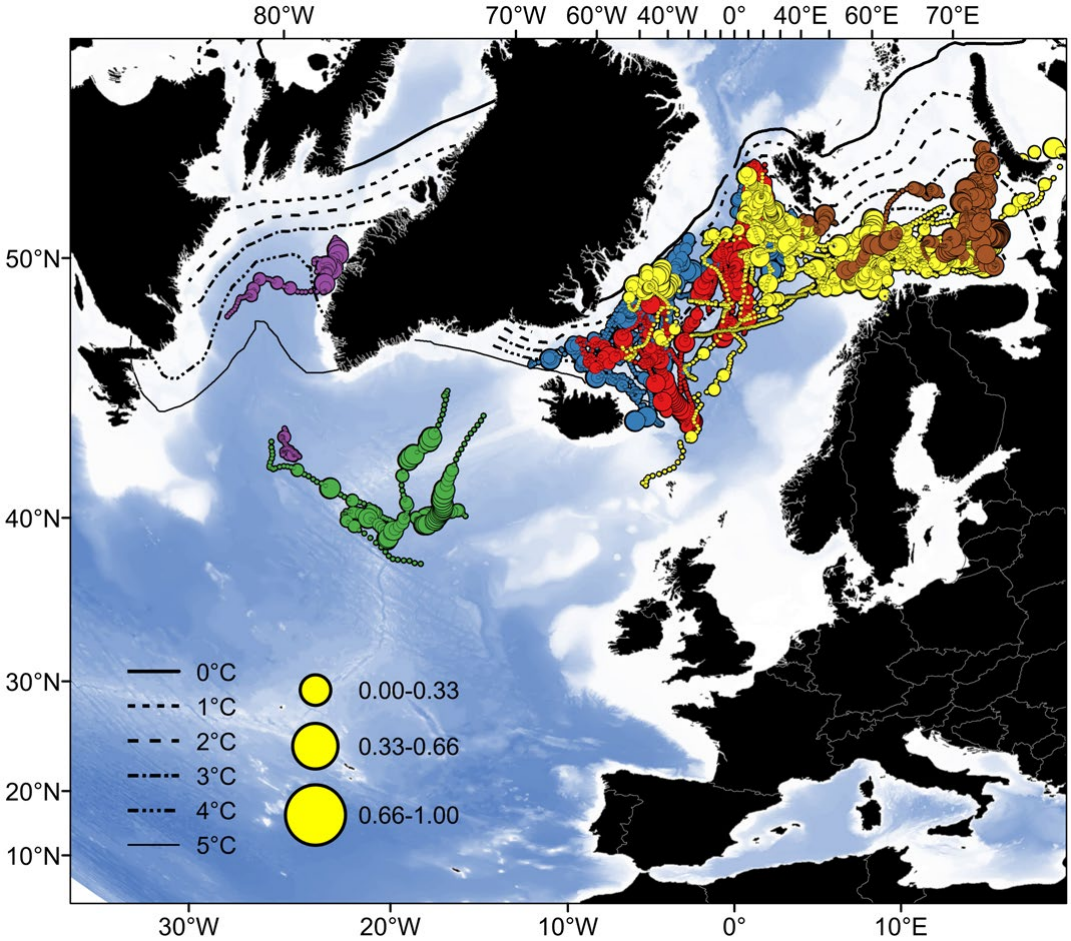
Post-migration diving behavior of Atlantic salmon in relation to the polar front (as bounded by the 0 °C and 5 °C annual mean temperature isolines). Post-migration is defined as > 11 weeks after release. Symbol size reflects the proportion of time spent at depths greater than 10 m. Color coding of salmon populations is the same as for Fig. 1A. Salmon released from Iceland and Spain provided data only from few fish and for a limited period, so data from these taggings are not included in the figure. Sea surface temperature contours were derived from mean monthly SST data 156 (NOAA_ERSST_V4) for the period of the study (January 2008 to December 2013). Data were provided by the NOAA/OAR/ESRL PSL, Boulder, Colorado, USA, from their Web site at 158 https://www1.ncdc.noaa.gov/pub/data/cmb/ersst/v4/netcdf/. Maps were drawn using ESRI ArcGIS Desktop v10.5.

Tagged salmon were predominately surface oriented, spending 80% of the time at depths less than 10 m, with occasional dives to greater depths (population-specific maximum depths from 220 to 870 m, Table 1). The frequency of dives to depths greater than 10 m increased for the southernmost populations during their post-migration period (i.e*.*, when they had reached the most distant areas from their home river) (Fig. 3). For the north-Norwegian populations this pattern was less clear, and the salmon initiated frequent diving soon after they had entered the open ocean.

**Figure 4:**
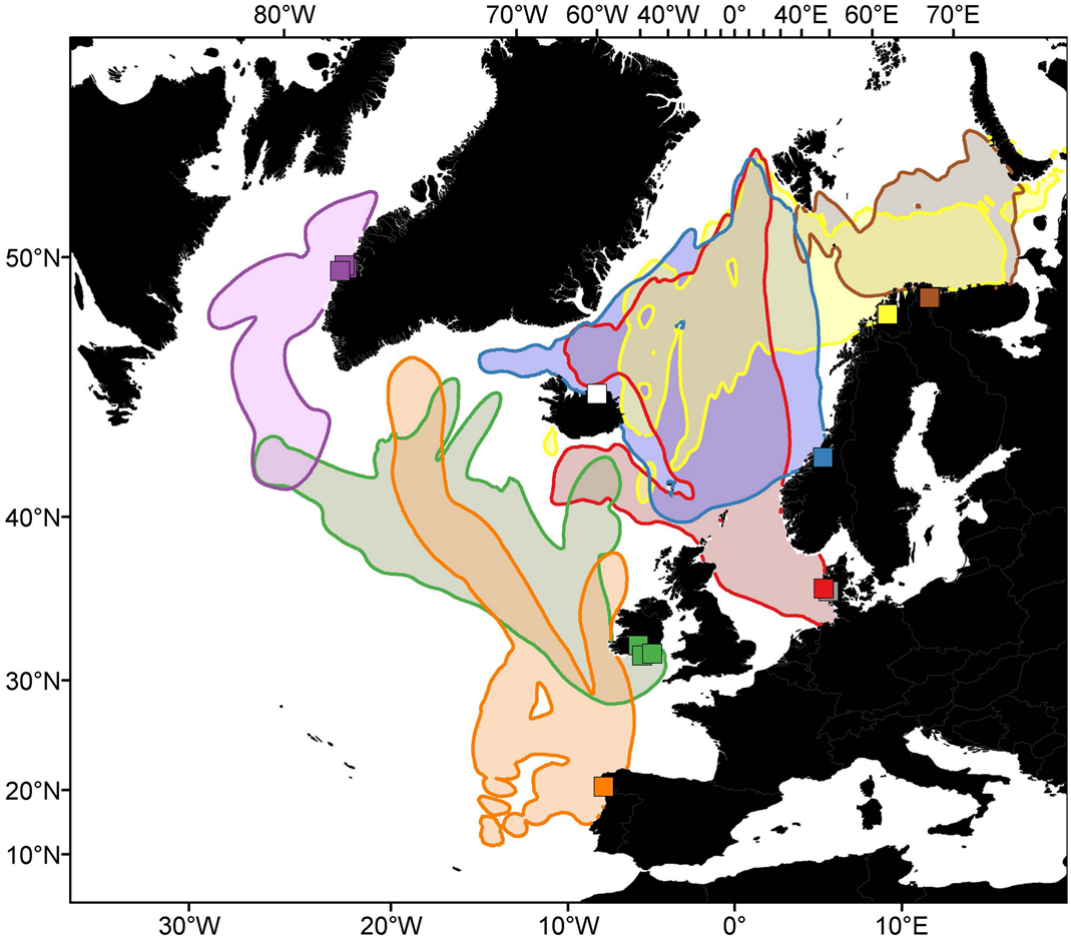
Area use during the ocean migration of tagged Atlantic salmon, shown with lines and shades with colors representing salmon from seven different areas (same color codes as in Fig. 1, with the two populations from Denmark combined due to the proximity and similarity of movements for fish of these rivers). Data from salmon tagged in Iceland were not included due to a small sample size. The area use is based on combined residency distributions for all fish from each population. Temporal area use is not considered here. The more detailed spatial intensity information within each population distribution is shown in Supplementary Figure S2. Maps were drawn using ESRI ArcGIS Desktop v10.5.

The overlap in the area use during the ocean migration varied substantially among the different populations, with greater overlap in ocean distribution between geographically proximate than distant populations (Fig. 4; Table 2). Populations from Denmark and Norway had the greatest overlap in distribution areas, with the northeast Norwegian population being the exception, with no overlap with Danish populations. Also, the populations from Spain and Ireland had a relatively large overlap in their distribution area. The Spanish and Irish fish did not overlap in distribution with Norwegian fish, and only to a small extent with Danish fish. The North American fish tagged at Greenland only overlapped to a small extent with the Irish fish (Fig. 4; Table 2). Temporal area use is not considered here, and the extent of overlap in area use among populations may be smaller if considering the time of the season different populations spend in different areas.

**Table 2:** Overlap in area use during the ocean migration between Atlantic salmon tagged in seven different areas, based on overlap in population-specific residency distributions. The values in the table represent Bhattacharrya’s affinity, which can be interpreted as proportion overlap (1 = total overlap, 0 = no overlap). Data from salmon tagged in Iceland were not included due to a small sample size.

	NE Norway	NW Norway	M Norway	Denmark	Ireland	Spain	Greenland
NE Norway	1.0	0.5	0	0	0	0	0
NW Norway	0.5	1.0	0.38	0.29	0	0	0
M Norway	0	0.38	1.0	0.58	0	0	0
Denmark	0	0.29	0.58	1.0	0.02	0	0
Ireland	0	0	0	0.02	1.0	0.32	0.05
Spain	0	0	0	0	0.32	1.0	0
Greenland	0	0	0	0	0.05	0	1.0

**Figure 5:**
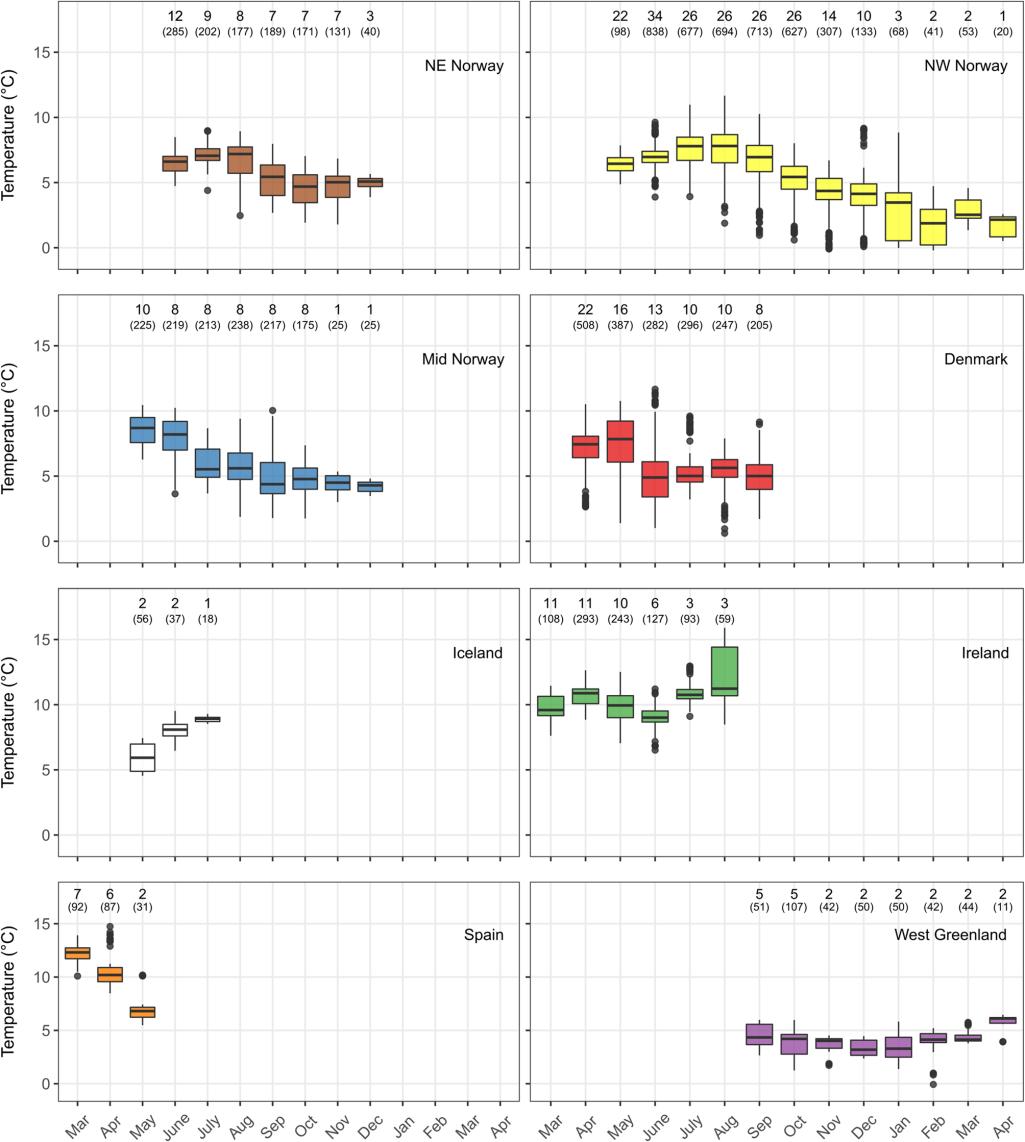
Thermal niche of tagged Atlantic salmon from different areas shown as temperature where they resided each month during the ocean migration. Numbers above each box show the number of individuals and the numbers in parentheses show the number of temperature recordings. Color codes are the same as in Fig. 1, with the two populations from Denmark combined due to the proximity and similarity of movements for fish of these rivers.

Thermal experience of fish from the different populations (Fig. 5) reflected the composition of the water masses encountered from the start of their migration and towards or along the polar front areas. Salmon from Denmark and Norway occupied cool northern Atlantic and/or Barents Sea water (daily averages from 0 to 11 °C depending on time of year). In contrast, salmon from the Irish, Spanish and Icelandic populations exploited generally warmer waters (5–16 °C) towards the areas south of Greenland in the western branch of the North Atlantic current. Maiden North American salmon tagged at Greenland occupied cool water (0–7 °C) in the West Greenland current and within the same range as the Norwegian populations during the same autumn and winter months (Fig. 5). The coldest winter temperatures experienced was by the north-western Norwegian population (average around 2 °C during February–April). However, this population and the salmon tagged at West Greenland were the only fish with data from this period.

## Discussion

Our study extends the known geographic area used by salmon during their migration in the North Atlantic Ocean and Barents Sea as reported by earlier studies based on conventional tagging and sampling surveys^15,16,20^. An extended use of the North Atlantic Ocean and Barents Sea was also suggested in recent studies using archival tags^12,23–26,28^, but these studies have concentrated on single populations or been restricted by low sample sizes. The present study indicated that multiple individuals from the Norwegian and Danish populations survived to migrate northward from their home river and reached latitudes as high as 80° N. This is to our knowledge the furthest north any Atlantic salmon has ever been recorded, extending previously assumed northern limits^8,30^. These results confirm that the foraging areas of Atlantic salmon currently extend to more northerly latitudes than previously thought. For populations in Denmark and Norway, the marine distribution is probably limited by the northern boundary of Atlantic currents. In contrast, the populations from Iceland, Ireland and Spain did not travel as far north, but instead crossed the main North Atlantic current and migrated towards southern Greenland, indicating a difference in ocean distribution for these populations. The less directed migration displayed by most of the North American salmon tagged at Greenland was likely due to these fish already being present at their assumed main ocean feeding grounds at the west coast of Greenland^15^ when tagged.

Despite the fact that salmon from different areas used different migration routes and ocean areas, they consistently migrated to and aggregated in assumed highly productive areas at the boundaries between large-scale frontal water masses where branches of the North Atlantic current lie adjacent to cold polar waters^31^. In these areas, previous analyses demonstrated frequent diving activity by tagged individuals^28^. The duration and diving profile of these dives suggested foraging behaviour, rather than predator escape, because the dives were U-shaped, typically lasted a few hours, and diving depths were related to the depth of the mixed layer during the different seasons^28^. Thus, the increased diving frequency is most likely an indication of increased feeding activity, emphasizing the importance of these productive regions as feeding areas for Atlantic salmon. In contrast to Atlantic salmon from the other areas, the two northernmost populations displayed a high diving frequency close to the shore immediately after sea entrance, as also shown by Hedger et al.^28^. These rivers are located closer to the frontal water masses, and these fish may have started extensive feeding earlier in their sea migration. This assumption is further supported by a study of Norwegian post-smolts, where the northernmost populations were feeding more extensively just after leaving their rivers than fish from southern populations^32^. Thus, the northern populations may benefit from a shorter migration route to the main feeding areas for salmon. However, given that many kelts are in poor condition when they enter the sea, it is likely that tagged fish from all populations were feeding pelagically in the first weeks at sea during the transit away from the coast when prey were available.

Migration from the rivers to the assumed foraging areas (i.e., the most distant areas they migrated to) was fast and direct for individuals from southern populations, while salmon from the northern Norway did not display similar direct migration routes. Our results are similar to those reported by an earlier study^26^ on the same North-Western Norwegian population as in the present study, and are likely related to the greater proximity to ocean frontal areas and rich food resources.

The results in the present study may have been influenced by the relatively large size of the tag compared to the size of the fish. Hedger et al.^33^ assessed tagging effects of PSATs on post-spawned Atlantic salmon by comparing their behaviour with salmon tagged with much smaller archival tags. They found that the overall depth distribution, ocean migration routes based on temperature recordings and return rates did not differ between salmon tagged with PSATs and smaller archival tags and concluded that PSATs are suitable for use in researching large-scale migratory behaviour of adult salmon at sea. However, salmon with PSATs dived less frequently and to slightly shallower depths^33^. Based on this, we believe the conclusions of the present study are valid despite potential tagging effects, but the diving depths and frequencies might be underestimated compared to non-tagged fish.

Diet data from adult salmon in the ocean are limited but show that salmon feed on a variety of prey taxa. Typically, herring (*Clupea harengus*), sand eels (*Ammodytes* spp.), capelin (*Mallotus villosus*) and myctophids dominate as fish prey, while euphausiids and amphipods often dominate as crustacean prey^34–36^. Although there exist some data of adult herring and capelin during parts of the year, there is limited information on the spatial and temporal distribution of crustaceans in these ocean areas, and it is therefore difficult to relate the salmon diving behaviour to availability of all their main prey items. However, salmon appeared to be able to forage on prey far below the surface, indicated by the frequent dives, and salmon at sea have also previously been shown to feed on the mesopelagic community^37,38^. Hedger et al.^28^ found that the diving depth increased with the depth of the mixed layer and hypothesised that stratification affected the aggregation of prey and thereby the salmon diving behaviour. They also showed that when the stratification disappeared during the dark winter months, the salmon dived less but their dives were deeper. Nevertheless, the possibilities to feed at different depths^28^, expand the foraging niche of salmon compared to feeding merely near the surface.

Dadswell et al.^11^ published the “merry-go-round hypothesis”, which implies that both first-time migrants and previous spawners from all salmon populations enter the North Atlantic Subpolar Gyres and move counter clockwise within these gyres until returning to their natal rivers. Although the full migration from river outrun to return was not followed in the present study (most tags popped off half-way into the migration), and some individuals indicated a counter clockwise migration pattern, most of the populations and individuals in this study clearly did not follow the North Atlantic Subpolar Gyres during the first months at sea. Therefore, most of our data did not support the merry-go-round hypothesis. However, some individuals from northern Norway seemed to follow the currents to a larger extent than individuals from other populations during the first months at sea. Previous studies on Atlantic salmon from Canada also documented that adults migrated either independently or against prevailing currents while at sea, indicating that the horizontal movement of adults are primarily governed by other factors^12,24^.

Due to the size limit of the pop-up-tags, we primarily tracked large post-spawned individuals that are more mobile than smaller first-time migrants. Although some studies have shown that first-time migrants can be found in the same areas as post-spawners from the same populations^8,30^ is not known to which extent the migration pattern and distribution of post-spawners represent the same migration pattern of first-time migrants. Due to a larger body size, it is possible that the migration of post-spawners depends to a lesser degree on ocean currents and gyres than do the movements of first-time migrants, especially in the first part of the migration. For example, we observed that the Irish and Spanish post-spawned individuals all crossed the main North Atlantic current towards Greenlandic waters. However, Irish and other southern European post-smolts have frequently been captured in the Norwegian Sea^20^, indicating that some of these individuals migrate and follow the main ocean current in a northward direction. It is possible that many of these post-smolts later migrate southwest towards Greenland and feed in these waters as maiden salmon before they return to rivers. This corresponds to the observation that it is mostly large (two sea-winter) southern European salmon (including Irish individuals) that are found in the southern Greenland feeding areas^20^. Therefore, it might be that the post-spawned salmon from these populations return to their primary feeding areas where they were feeding as maiden salmon from their first sea migration, and not necessarily to the same area as they started their feeding migration as post-smolts.

Populations differed in their ocean distribution, but the distribution also overlapped to some degree between or among populations, with more overlap between geographically proximate than distant populations. Some populations never overlapped in geographical distribution during the study. The populations from Ireland and Spain did not overlap with the Norwegian and Danish salmon, but there was a small spatial overlap between the Irish salmon and the North American salmon tagged at Greenland, although area use by these populations did not overlap in time. It is known that populations from North America and Europe largely use different parts of the North Atlantic, with more North American salmon in the western part and more European salmon in the eastern part of the ocean although they have been shown to mix at the feeding grounds at the Faroes and at Greenland^12,15,16,18,20^. For the Spanish population, it should be noted that tagged individuals were followed for a relatively short period, and a larger sample size over a longer period might have shown some overlap with the northern European populations, based on the initial northward direction of two individuals. At the same time as populations differed in their ocean distribution, there were also relatively large within-population differences in migration routes and geographic distribution. Individual differences in migration routes and ocean distribution of salmon from the same population, even within the same year, were also shown by Strøm et al.^12,26^. Collectively, these results imply that salmon from different populations will experience highly different ecological conditions, potentially contributing to between-and within-population variation in growth and survival. Since our data are limited by a varying number of individuals among the studied populations, and restricted mainly to post-spawned salmon, our results represent a minimum overlap among the populations so the actual overlap may be larger. Nevertheless, this strongly indicates a varying degree of geographical separation in ocean feeding areas. Thus, geographically close populations will to a larger extent be influenced by similar conditions in the ocean than more distant populations.

The study was carried out over several years, with not all sites having tagging undertaken in the same years. There is a possibility that geographic area use and overlap among populations may vary among years, according to variation in environmental conditions among years^29^. However, data from multiple years for some populations suggest consistent population specific migration routes and area use among years, indicating that the principal patterns are stable over time for particular salmon populations.

The differing distributions of salmon from particular populations in different oceanic regions might simply be a function of distance to appropriate feeding grounds from the different home rivers, with individuals from the different rivers mainly adapted to seek the closest feeding areas. The route selection during the migration might in addition be a result of each individuals’ opportunistic behaviour and which food resources and environmental conditions they encounter along the journey. As discussed above, the experience and learning during the first ocean migration might also impact individuals’ route choice and area use. Salmon from southern populations used more southern ocean areas, and hence stayed in warmer water, than salmon from the northern populations. We cannot rule out that salmon from different populations have different temperature preferences due to different thermal selection regimes in their home rivers, but similar to a previous study^29^, we suggest that the differences in thermal habitat among populations utilising different areas at sea are mainly driven by availability of prey fields. There is generally little support for the hypothesis that variation in salmonid growth rates reflects thermal adaptations to their home stream^39^.

Despite the variation in migration patterns among and within populations, most individuals seemed to migrate to distant ocean frontal areas. This suggests that climate change may have greater impact on populations originating further south, because the distances and time required to travel to feeding areas will increase if the boundary between Atlantic and Arctic waters move northward because of ocean warming. Our study has shown that several populations are able to migrate over large distances, but the capacity for populations to adapt to an increased migration distance is unknown. Given increased migration time, especially for southern populations, the time available for accumulating important energy reserves will likely be reduced. In addition, increased water temperatures in the North Atlantic may also increase the energy expenditure that the individual fish spend per unit of distance when migrating from their home rivers towards the feeding areas. This may affect all populations to some degree, and may contribute to an additional burden for Atlantic salmon populations that are already in a poor state. This will also add to the hypothesized negative effect of climate change in freshwater for the southern populations, where temperatures will have a greater likelihood of reaching to growth inhibiting levels compared to more northern populations^39^.

Taking advantage of the development of electronic tags, we have shown an extended use of the North Atlantic Ocean by Atlantic salmon, including the Barents Sea, which contrasts to the earlier strong focus on feeding areas at the Faroes, West Greenland and in the Norwegian Sea in previous studies. These results expand the knowledge on the marine foraging and habitat niche of Atlantic salmon, in terms of geography, migration behaviour and thermal niche. The existence of feeding areas at the boundaries between Atlantic and Arctic surface currents suggests that salmon have a strong link to Arctic oceanic frontal systems. We have further shown that salmon from different populations may migrate to different ocean frontal areas in the North Atlantic Ocean and Barents Sea and therefore be impacted by different ecological conditions that may contribute to within-population variation in growth and survival. We also conclude that climate induced changes in oceanographic conditions, which will likely alter the location of and distance to polar frontal feeding areas, may have region-specific influences on the length and cost of the Atlantic feeding migrations, particularly affecting the southern populations most. As the polar oceans get warmer and current patterns shift, changes in the location and productivity of high latitude fronts will become evident. As migration distances become longer, or more variable, and the time accumulating energy is reduced, the variation in the marine survival and productivity of different populations are likely to become more marked. Combined, our results help to shed light on important ecological process that shape Atlantic salmon population dynamics within most of its distribution area.

## Material and methods

### Capture and handling of Atlantic salmon for tagging

Atlantic salmon post-spawners from seven European areas were tagged with pop-up satellite archival tags (n = 204, Table 1). The sites included north-eastern Norway (River Neiden), north-western Norway (River Alta), Mid Norway (River Orkla), Denmark (River Skjern and Varde), Ireland (Rivers Blackwater, Suir, Nore and Barrow), Spain (River Lérez) and Iceland (River Laxa). In addition, maiden salmon captured and tagged at Greenland (Nuuk) were included to provide information on Atlantic salmon in the north-western Atlantic Ocean. The Greenland fish were all of North American origin, as confirmed by genetic analyses^40^. Salmon post spawners were caught in rivers during spring by electrofishing (Denmark), in a trap (Spain) or by angling (Norway, Ireland and Iceland) and placed in a net pen in saltwater (about 5 × 5 × 5 m) (Norway, Ireland) or in a freshwater tank close to the river mouth for 1–2 weeks (Denmark and Iceland). The Spanish salmon were kept in a circular freshwater tank (3 m in diameter, 1.5 m deep) for up to 3 months before tagging.

The largest individuals were selected for tagging due to the size of the tags. The maiden salmon at Greenland were captured in constantly tended gillnets (n = 19), trap nets (n = 4) or rod (n = 1). Upon capture, fish were transferred to a 400 l water tank on board a boat, where they were tagged and released post-recovery close to the capture location.

### Tags, tagging and release

The pop-up satellite archival tags (PSAT model X-tag, size 120 × 32 mm plus 185 mm long antenna, 40 g in air, Microwave Telemetry Inc. Columbia, Maryland, USA) were slightly buoyant and programmed to release from the fish on a certain date or when the fish had remained at a constant depth for 4 or 5 days (in the case of fish mortality, or the tag attachment was expelled from the fish). Once at the surface, the tag location was received by ARGOS-satellites, and the tag transmitted a subset of archived data (depth, temperature and estimates of sunset/sunrise times) to the satellites (http://www.argos-system.org), with a temporal resolution of 15–60 min depending on the deployment duration.

Before tagging, the fish were anaesthetised by using MS-222 (Ireland, Iceland and Greenland), benzocaine (Denmark and Spain), or 2-phenoxyethanol (Norway) and brought to a tagging cradle where body length and mass were measured. The tags were attached externally to individual fish by bridling the tag to two cushioned back plates that were wired through the dorsal musculature below the dorsal fin^28^. The tag mass averaged 0.57% of the fish body mass (range 0.40–0.74%). A numbered custom-made disc tag (http://www.floytag.com) with contact information was attached to one of the plates in case the fish was recaptured to test for tagging effects in a separate study^33^.

After tagging, the fish were placed in a large tank or cage and released as soon as they appeared recovered, e.g*.*, breathing and swimming normally and reacting when tactile pressure was applied to the tail base. Since the tags would release if they were at constant depth for 4–5 days we did not want the salmon to remain in the rivers with homogeneous depth after tagging. Therefore river-captured salmon were transported 5–15 km away from the river mouth before release.

The experimental procedures were in each case approved by national authorities. All methods were carried out in accordance with relevant guidelines and regulations and fish tagging was approved by the Norwegian Animal Research Authority, the Animal Experimentation Council of Denmark (incl. Greenland), The Icelandic Animal Research Authority, the Department of Health and Children Ireland, and the Ponteverda Province Spain.

### Data recovery and analysis, reconstruction of ocean migration

In total, data from 148 Atlantic salmon from a total of ~ 15,000 days were recovered (Table 1). Average data recovery from the PSATs was 69% of the data that could potentially have been recovered. Migration tracks from release of the fish after tagging until the tag popped up could be estimated for 105 salmon based on daily geographic location estimates (Fig. 1). The pop-up location of the tag was provided for 43 additional salmon (Fig. 1).

### Data analyses

Geolocation of tagged fish to reconstruct oceanic migration was done using hidden Markov models (HMMs). HMMs are state space models that generate a series of non-parametric spatial probability distributions by a forward–backward recursion on a defined spatial grid^41^. HMMs enable geolocation of animals in scenarios when light-based geolocation estimates are either poor or absent^41^.

The HMM developed by Pedersen^41^ was the primary model used for geolocation. The results were compared to outputs generated by using the method described by Strøm et al.^12^ to quantify potential biases in estimated migration route caused by the structure of the geolocation models. In both models, daily data likelihoods were calculated using the estimated location (latitude and longitude), mean daily temperature at the surface (< 20 m) and maximum depth^12,41^. The only major difference between the two models was the spatial grid defining the model domain, resulting in some structural difference in the underlying random walks. In the approach developed by Pedersen^41^ the spatial grid was discretised based on latitude and longitude, whereas in the method used by Strøm et al.^12^, the grid was discretised based on distances. In both models, individual migration routes were reconstructed using the mean of 1000 random tracks^42^. The comparison of outputs between the two models showed little differences between individual migration trajectories, indicating that the outputs were not affected by the spatial structure of the models.

Population-specific ocean distributions were quantified by calculating the combined residency distributions (RDs) for all fish with ocean migrations exceeding 10 days. RDs are the cumulative probability distribution for the entire spatial domain and provide an estimate of the ocean distribution that includes the uncertainty of the data^41^. The spatial overlaps among populations were determined by calculating the overlap in population-specific RDs using Bhattacharrya’s affinity^43^. Icelandic data were too few to be included. Data from the two Danish populations were combined in temporal and annual comparisons, because they belong to rivers close to each other. Depth data for salmon from Rivers Alta, Neiden and Orkla in Norway have previously been analysed in relation to day length and temperature stratification by Hedger et al.^28^, but not in relation to their modelled migration routes as in the present study.

## Supplementary Information

Supplementary Information.

## Data Availability

Data will be made available upon request.
