# Supplementary material for: Redefining the oceanic distribution of Atlantic salmon
Source: Sci Rep. 2021 Jun 10;11:12266. doi: 10.1038/s41598-021-91137-y (PMC8192511; doi:10.1038/s41598-021-91137-y)
Supplement: Supplementary file 1 — Supplementary Information. [file 41598_2021_91137_MOESM1_ESM.pdf]

Supplementary material:

Redefining the oceanic distribution of Atlantic salmon

Audun H. Rikardsen[1,3*], David Righton[2], John Fredrik Strøm[1], Eva B. Thorstad[1,3], Patrick Gargan[4], Timothy Sheehan[5], Finn Økland[3], Cedar M. Chittenden[1], Richard D. Hedger[3], Tor F. Næsje[3], Mark Renkawitz[5], Johannes Sturlaugsson[6], Pablo Caballero[7], Henrik Baktoft[8], Jan G. Davidsen[9], Elina Halttunen[1], Serena Wright[2], Bengt Finstad[10], Kim Aarestrup[8]

Author affiliations

[1]*UiT The Arctic University of Norway, Department of Arctic and Marine Biology, NO-9037 Tromsø, Norway.*

[2]*Centre for Environment, Fisheries and Aquaculture Science, Lowestoft, UK.*

[3]*Norwegian Institute for Nature Research, 9007 Tromsø/7034 Trondheim, Norway.*

[4]*Inland Fisheries Ireland, 3044 Lake Drive, Citywest Business Campus, Dublin 24, Ireland.*

[5]*NOAA Fisheries Service, Northeast Fisheries Science Center, Woods Hole, MA, USA.*

[6]*Laxfiskar, Mosfellsbaer, Iceland.*

[7]*Servicio de Conservación de la Naturaleza de Pontevedra, Ponteverda, 36071, Spain.*

[8]*Technical University of Denmark, National Institute of Aquatic Resources, Silkeborg, Denmark.*

[9]*NTNU University Museum, Department of Natural History, NO-7491 Trondheim, Norway.*

[10]*NTNU University of Science and Technology, Department of Biology, NO-7491 Trondheim, Norway.*

* Corresponding author; audun.rikardsen@uit.no

Supplementary figures and tables:

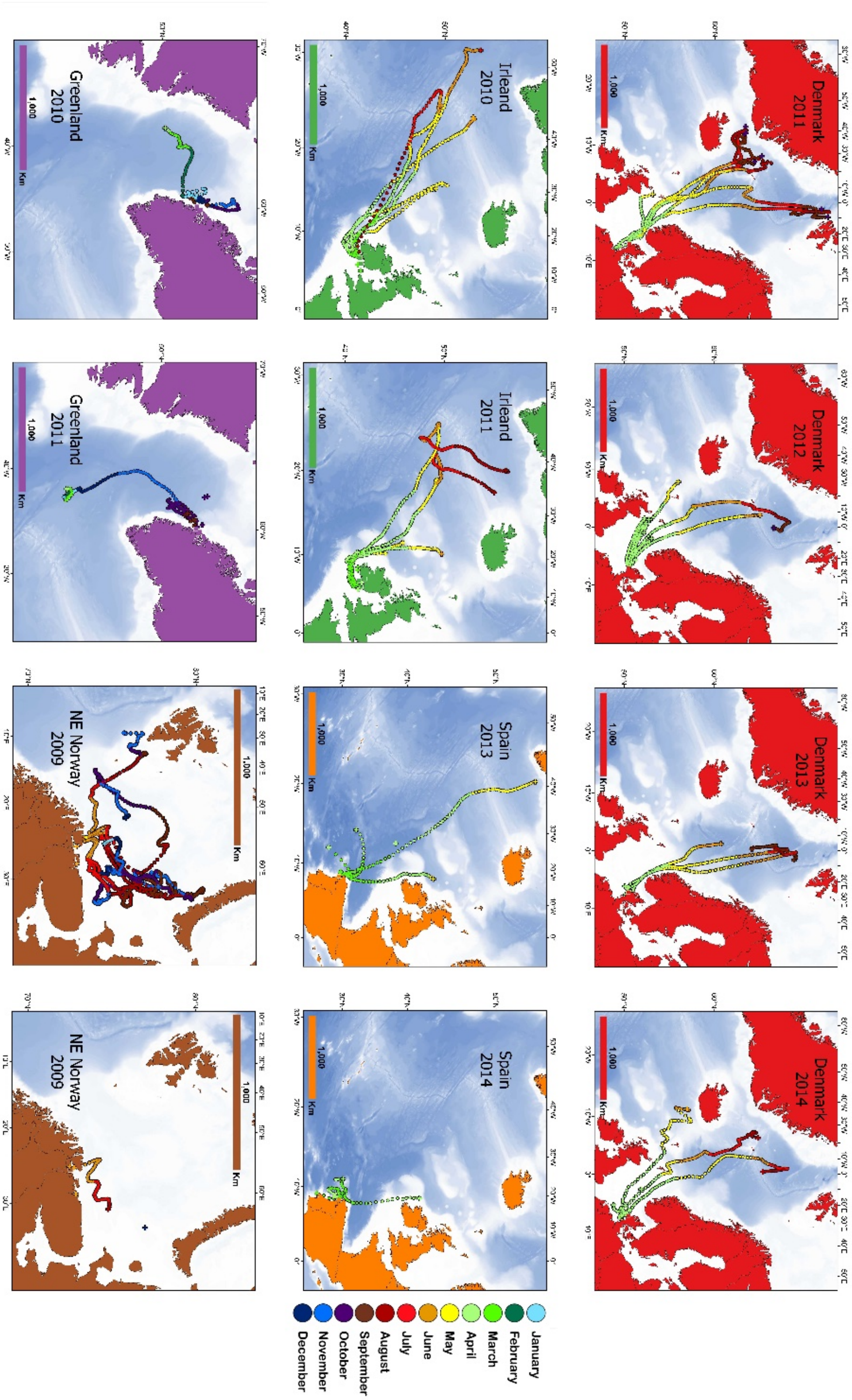

Figure S1. Migration tracks by month (colour coded) of individual Atlantic salmon in populations tagged in multiple years (except for NW Norway, which is shown in Figure 2). Each panel shows the tracks of fish tagged in one geographic area (indicated by the colour of the land masses in each map, corresponding to colour codes in Figure 1). Darker blue shading in the ocean indicates increasing depth[44]. Maps were drawn using ESRI ArcGIS Desktop v10.5.

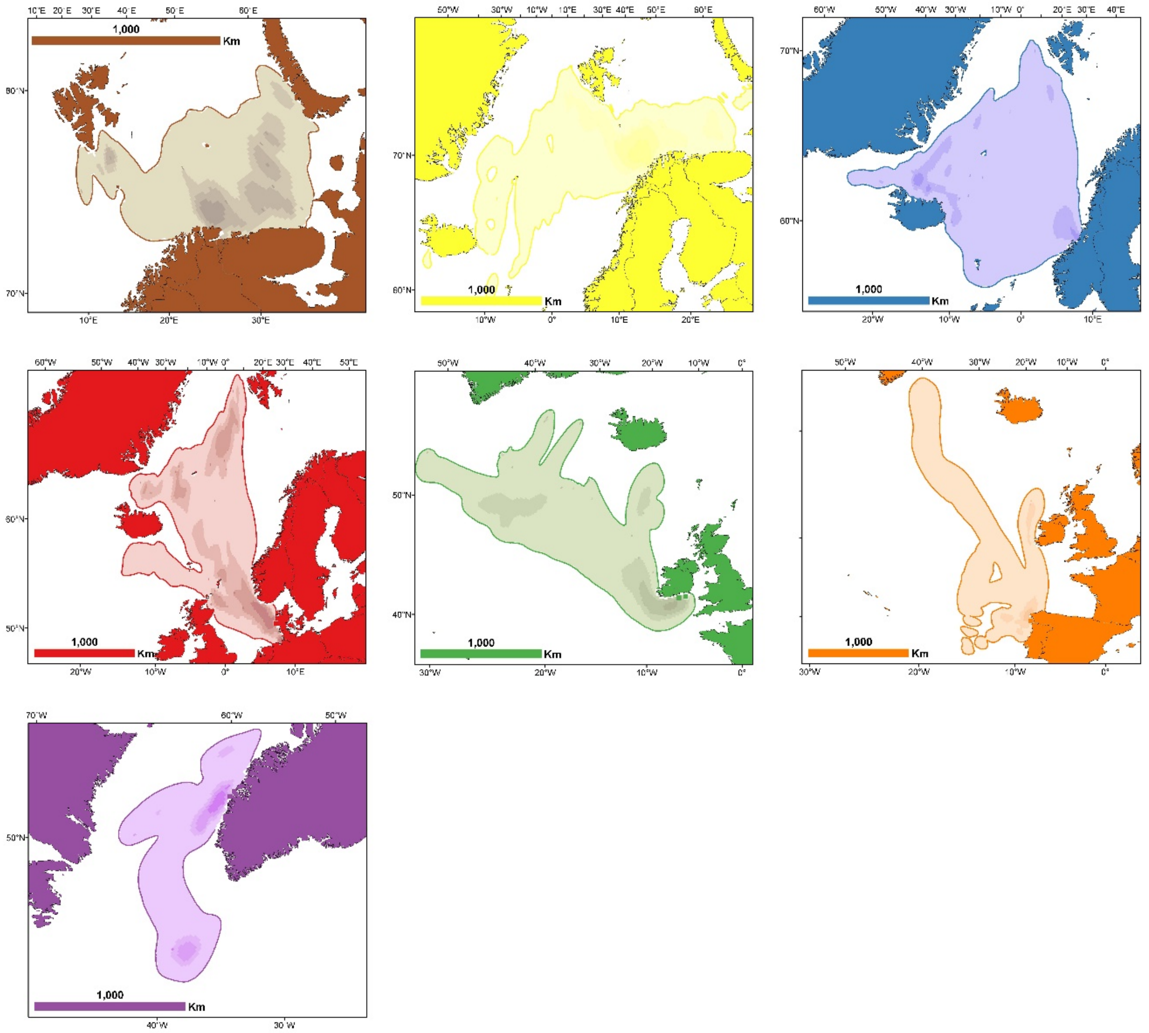

Figure S2. Area use and spatial intensity information within each population distribution during the ocean migration of tagged Atlantic salmon, shown with lines and graded shades (to indicate intensity) with colors representing salmon from seven different areas (same color codes as in Figure 1, with the two populations from Denmark combined due to the proximity and similarity of movements for fish of these rivers). Data from salmon tagged in Iceland were not included due to a small sample size. The area use is based on combined residency distributions for all fish from each population. Temporal area use is not considered here. The overlap between these populations is shown in Figure 4. Maps were drawn using ESRI ArcGIS Desktop v10.5.

Table S1. Biometric data of tagged individual post-spawned Atlantic salmon that provided enough data to be included in the study. This includes fishes from eleven rivers in eight geographic areas in the north-east Atlantic and maiden salmon captured off Greenland. Each fish is given with tag ID#, release and pop-off location information, maximum distance covered in a straight line away from the release sight (Dist. km), maximum depth recorded (max depth m), number of days the tag was recording (Days record) and percentage of stored data retrieved from the tag (% data transm.). For fish with available and useable scale samples, the smolt age and year at sea was cannulated. Year at sea represent number of feeding seasons (spring-autumn) spent at sea, and the first number gives the first number of feeding seasons at sea, while the + numbers give the additional ocean feeding seasons as repeat spawners.

ID #	Contry	Population	Release Lat N	Release Long E	Length (cm)	Weight (kg)	Smol age	Year at sea	Release date	Pop-off date	Days record	Pop-off Lat N	Pop-off Long E	Dist (km)	Max depth (m)	% data transm	Reason pop-off
83100	Norway (NW)	Alta	70,0	23,2	100,5	7,6	4	3	22.5.08	15.10.08	146	69,5	38,1	631	302	87 %	Const. press.
83101	Norway (NW)	Alta	70,0	23,2	111	9,9	3	3+1+1	22.5.08	19.11.08	181	73,6	30,5	472	459	78 %	Const. press.
83102	Norway (NW)	Alta	70,0	23,2	105	8,5	4	3+1	22.5.08	19.6.08	28	72,2	23,1	246	184	100 %	Const. press.
83104	Norway (NW)	Alta	70,2	23,0	109,5	9,7	4	3+1+1	22.5.08	8.10.08	139	70,2	23,0	-	421	100 %	Recovered
83105	Norway (NW)	Alta	70,0	23,2	96	6,7	4	3	22.5.08	28.10.08	159	75,8	0,5	974	432	1 %	Const. press.
83106	Norway (NW)	Alta	70,0	23,2	109,5	9,7	4	3+1+1	22.5.08	12.6.08	21	73,0	20,6	345	146	100 %	Const. press.
83108	Norway (NW)	Alta	70,0	23,2	94,5	5,9	3	3	22.5.08	2.7.08	41	70,0	23,2	-	54	100 %	Recovered
83109	Norway (NW)	Alta	70,0	23,2	93,5	6,7	4	3	22.5.08	24.6.08	33	70,1	15,5	286	281	100 %	Const. pressure
71067	Norway (NW)	Alta	70,0	23,2	94	6,1	4	3	29.5.09	1.11.09	156	73,8	25,4	436	446	77 %	On time
71068	Norway (NW)	Alta	70,0	23,2	91,5	5,6	4	3	29.5.09	19.11.09	174	72,1	1,1	828	483	4 %	On time
71069	Norway (NW)	Alta	70,0	23,2	97	6,6	5	3	29.5.09	1.11.09	156	78,9	5,0	1117	456	66 %	On time
71070	Norway (NW)	Alta	70,0	23,2	104,5	7,9	4	3+1	29.5.09	1.11.09	156	70,4	51,7	1076	301	43 %	On time
71071	Norway (NW)	Alta	70,0	23,2	101,5	7,9	3	3+1	29.5.09	1.11.09	156	74,0	30,3	512	408	75 %	On time
71072	Norway (NW)	Alta	70,0	23,2	101,5	8	4	3+1	29.5.09	1.11.09	156	75,6	8,2	788	521	77 %	On time
71073	Norway (NW)	Alta	70,0	23,2	96	7,2	4	3	29.5.09	1.11.09	156	73,7	32,6	528	344	69 %	On time
71075	Norway (NW)	Alta	70,0	23,2	100	6,7	4	3	29.5.09	18.6.09	20	72,9	15,5	419	-	0 %	Const. press.
71076	Norway (NW)	Alta	70,0	23,2	92,5	6,9	4	3	29.5.09	17.7.09	19	70,5	23,7	55	32	46 %	Const. press.
83161	Norway (NW)	Alta	70,0	23,2	100	7,9	3	3	29.5.09	1.11.09	156	73,8	23,6	430	354	64 %	On time
83163	Norway (NW)	Alta	70,0	23,2	96	6,7	4	3	29.5.09	1.11.09	156	71,8	41,9	715	333	66 %	On time
83164	Norway (NW)	Alta	70,0	23,2	96	6,1	4	3	29.5.09	20.11.09	175	75,7	13,6	711	-	0 %	On time
83165	Norway (NW)	Alta	70,0	23,2	98	7,5	3	3	29.5.09	15.11.09	174	75,1	13,4	593	494	64 %	Const. press.
89302	Norway (NW)	Alta	70,0	23,2	93	6,1	4	3	29.5.09	14.12.09	204	74,1	8,4	666	413	76 %	Const. press.
89318	Norway (NW)	Alta	70,0	23,2	109	9,6	-	3+1	29.5.09	8.12.09	197	79,6	8,0	1090	376	59 %	Const. press.
89319	Norway (NW)	Alta	70,0	23,2	96,5	6,7	5	3	29.5.09	27.6.09	29	71,4	17,0	272	408	100 %	Const. press.
89320	Norway (NW)	Alta	70,0	23,2	96,5	6,1	4	2+1	29.5.09	8.12.09	197	73,4	19,8	391	446	75 %	Const. press.
35087	Norway (NW)	Alta	70,0	23,2	95	6,2	4	4	24.5.10	2.11.10	164	76,0	15,4	685	456	66 %	Const. press.
35109	Norway (NW)	Alta	70,0	23,2	95	6,4	5	4	24.5.10	12.12.10	202	75,6	11,7	652	634	47 %	Const. press.
35229	Norway (NW)	Alta	70,0	23,2	109	7,9	-	-	24.5.10	25.12.10	218	70,8	31,8	339	279	60 %	Const. press.
35230	Norway (NW)	Alta	70,0	23,2	105,5	8,3	4	3+1+1	24.5.10	14.12.10	204	72,7	2,5	835	376	5 %	Too Deep
35247	Norway (NW)	Alta	70,0	23,2	95	6,2	4	3+1	24.5.10	2.11.10	162	77,2	2,5	989	21	0 %	Const. press.
35252	Norway (NW)	Alta	70,0	23,2	93	5,8	5	4	24.5.10	1.4.11	312	71,5	1,0	894	499	57 %	Popped on time
35259	Norway (NW)	Alta	70,0	23,2	101	7	5	3+1	24.5.10	2.12.10	192	73,7	22,0	416	419	44 %	Const. press.
35260	Norway (NW)	Alta	70,0	23,2	109	8,6	4	3+1+2	24.5.10	19.1.11	240	63,7	-20,9	2067	564	45 %	Const. press.
35261	Norway (NW)	Alta	70,0	23,2	97	7	5	3+1+1	24.5.10	20.4.11	331	70,0	23,2	1325	413	100 %	Recovered
35527	Norway (NW)	Alta	70,0	23,2	100,5	8,1	4	3+1+1	24.5.10	19.6.10	26	73,2	14,4	451	338	99 %	Const. pressure
35534	Norway (NW)	Alta	70,0	23,2	111,5	9,7	4	4+1+1	24.5.10	1.12.10	191	73,7	18,5	452	424	65 %	Const. press.
35536	Norway (NW)	Alta	70,0	23,2	96,5	7,4	4	4	24.5.10	6.1.11	227	75,8	2,8	-	-	0 %	Const. press.
35538	Norway (NW)	Alta	70,0	23,2	95	6,4	4	3	24.5.10	3.11.10	163	77,8	4,8	1007	403	27 %	Const. press.
35547	Norway (NW)	Alta	70,0	23,2	94	7,1	4	3	24.5.10	4.12.10	194	73,0	29,3	395	274	25 %	Const. press.
35548	Norway (NW)	Alta	70,0	23,2	99	7,2	4	4	24.5.10	13.12.10	203	73,6	14,4	476	612	61 %	Const. press.
35528	Norway (NW)	Alta	70,0	23,2	92	5,4	5	4	27.5.10	1.4.11	309	70,3	-6,2	1120	145	1 %	On time
35529	Norway (NW)	Alta	70,0	23,2	101	8,3	4	3+1	27.5.10	5.12.10	192	73,7	19,0	461	279	2 %	Const. press.
35530	Norway (NW)	Alta	70,0	23,2	96,5	6,7	4	3	27.5.10	14.11.10	171	72,1	46,4	869	193	29 %	Const. press.
35532	Norway (NW)	Alta	70,0	23,2	99	7,6	4	4	27.5.10	14.6.10	19	70,9	25,5	133	322	99 %	Const. press.
36078	Norway (NW)	Alta	70,0	23,2	93	6,2	4	3	27.5.10	11.6.10	16	71,7	14,9	355	188	98 %	Const. press.
36082	Norway (NW)	Alta	70,0	23,2	95,5	6,2	4	4	27.5.10	12.6.10	16	70,7	19,5	159	193	93 %	Const. press.
71077	Norway (NE)	Neiden	70,2	29,8	94,5	5,3	-	-	31.5.09	20.11.09	174	70,6	40,6	481	204	55 %	Const. press.
71078	Norway (NE)	Neiden	70,2	29,8	91	4,8	-	-	31.5.09	18.11.09	172	74,7	12,6	841	397	71 %	Const. press.
71079	Norway (NE)	Neiden	70,2	29,8	86,5	4,5	-	-	31.5.09	24.6.09	24	70,1	30,7	134	166	17 %	Const. press.
71080	Norway (NE)	Neiden	70,2	29,8	94,5	5,5	-	-	31.5.09	30.6.09	31	69,8	30,3	20	75	100 %	Const. press.
71081	Norway (NE)	Neiden	70,2	29,8	101,5	7,9	-	-	31.5.09	20.6.09	20	69,5	33,2	131	118	65 %	Const. press.
71082	Norway (NE)	Neiden	70,2	29,8	96,5	6,1	-	-	31.5.09	20.11.09	174	72,7	45,4	643	306	43 %	Const. press.
71083	Norway (NE)	Neiden	70,2	29,8	92	5,3	-	-	31.5.09	21.11.09	175	72,6	43,6	589	311	48 %	Const. press.
71084	Norway (NE)	Neiden	70,2	29,8	96	6,1	-	-	31.5.09	16.12.09	199	74,0	30,9	418	333	80 %	Const. press.
71085	Norway (NE)	Neiden	70,2	29,8	107	9,8	-	-	31.5.09	14.12.09	198	70,1	37,3	247	365	32 %	Const. press.
71086	Norway (NE)	Neiden	70,2	29,8	96	6,7	-	-	31.5.09	10.1.10	224	71,5	33,8	193	295	46 %	On time
36061	Norway (NE)	Neiden	70,2	29,8	92,5	5,8	3	3	30.5.10	7.6.10	8	69,9	29,4	38	220	7,00 %	Const. press.
36149	Norway (NE)	Neiden	70,2	29,8	73	2,9	3	3	30.5.10	6.8.10	69	68,9	41,5	473	107	84,34 %	Predation
36152	Norway (NE)	Neiden	70,2	29,8	80	3,8	4	3	30.5.10	24.6.10	25	69,3	33,8	181	86	65,40 %	Const. press.
36463	Norway (NE)	Neiden	70,2	29,8	86	4,1	3	3+1	30.5.10	27.12.10	211	71,3	45,9	-	-	0,00 %	Unknown
36464	Norway (NE)	Neiden	70,2	29,8	88	4,7	4	3+1	30.5.10	19.6.10	21	70,1	29,9	10	199	82,04 %	Const. press.
35550	Norway (M)	Orkla	63,6	10,0	93	5,9	-	4	5.5.10	1.11.10	180	69,0	-10,6	1092	440	100 %	Recovered
35650	Norway (M)	Orkla	63,6	10,0	114,5	9,8	3	4+1+1+1	5.5.10	30.5.10	25	67,7	11,2	460	113	99 %	Const. press.
35959	Norway (M)	Orkla	63,6	10,0	109	8,8	4	4+1+1+2	5.5.10	1.11.10	180	79,0	5,4	1721	505	43 %	On time
36019	Norway (M)	Orkla	63,6	10,0	95	6,5	4	4	5.5.10	1.11.10	180	67,0	-11,8	1075	317	76 %	On time
35549	Norway (M)	Orkla	63,6	10,0	92,5	5	4	4	6.5.10	31.5.10	25	63,7	9,6	22	38	70 %	Const. press.
35552	Norway (M)	Orkla	63,6	10,0	93	5,8	-	-	6.5.10	1.11.10	179	76,8	12,7	1478	644	57 %	Const. press.
35990	Norway (M)	Orkla	63,6	10,0	99	7	4	4	6.5.10	3.10.10	150	69,0	-8,0	993	247	55 %	Const. press.
36003	Norway (M)	Orkla	63,6	10,0	88,5	4,7	3	4	6.5.10	1.11.10	179	67,4	-10,3	1023	467	71 %	On time
36459	Norway (M)	Orkla	63,6	10,0	96	6,9	4	4	6.5.10	10.10.10	157	74,4	-2,9	1310	446	72 %	Const. press.
36460	Norway (M)	Orkla	63,6	10,0	95,5	6,2	4	5	6.5.10	28.12.10	236	65,0	-12,2	1068	526	61 %	Const. press.

Table S1. (Continuing)

ID #	Contry	Population	Release Lat N	Release Long E	Length (cm)	Weight (kg)	Smol age	Year at sea	Release date	Pop-off date	Days record	Pop-off Lat N	Pop-off Long E	Dist (km)	Max depth (m)	% data transm	Reason pop-off
49450	Denmark	Skjaerne	56,0	8,1	100	7,1	1	2+1	31.3.11	7.10.11	190	68,5	-23,1	2097	-	0 %	Unknown
49451	Denmark	Skjaerne	56,0	8,1	78,5	3	1	2	31.3.11	5.5.11	35	60,8	-2,9	862	97	64 %	Const. press.
49485	Denmark	Skjaerne	56,0	8,1	87	3,7	1	3	31.3.11	2.10.11	185	68,7	-21,4	2047	338	64 %	On time
49544	Denmark	Skjaerne	56,0	8,1	89	4,2	2	3	31.3.11	1.10.11	184	71,4	-15,9	2061	279	58 %	On time
49792	Denmark	Skjaerne	56,0	8,1	95	5,5	2	2+1	31.3.11	1.10.11	184	70,9	-13,8	1961	478	77 %	On time
49799	Denmark	Skjaerne	56,0	8,1	90	4,6	1	3	31.3.11	1.10.11	184	78,8	3,5	2548	462	69 %	On time
49800	Denmark	Skjaerne	56,0	8,1	76	2,7	1	3	31.3.11	10.5.11	40	58,0	5,6	267	215	65 %	On time
49806	Denmark	Skjaerne	56,0	8,1	94	5,1	1	3	31.3.11	1.10.11	184	79,4	8,1	2607	585	46 %	On time
115237	Denmark	Skjaerne	56,0	8,1	86	4,1	-	-	2.4.12	8.4.12	6	57,7	2,2	431	38	84 %	Died and sank
115238	Denmark	Skjaerne	56,0	8,1	92	4,5	-	-	2.4.12	27.4.12	25	59,0	0,9	580	75	67 %	Const. press.
115239	Denmark	Skjaerne	56,0	8,1	86	4	-	-	2.4.12	30.4.12	28	58,3	-2,4	692	75	88 %	Const. press.
115240	Denmark	Skjaerne	56,0	8,1	80	3,2	-	-	2.4.12	2.10.12	183	73,7	0,4	2001	312	59 %	On time
115241	Denmark	Skjaerne	56,0	8,1	80	3,2	-	-	2.4.12	27.4.12	25	59,6	-1,8	774	75	85 %	Const. press.
115242	Denmark	Skjaerne	56,0	8,1	86	3,7	-	-	2.4.12	14.5.12	42	62,0	-13,7	1387	145	86 %	Predation
115243	Denmark	Skjaerne	56,0	8,1	83	3,3	-	-	2.4.12	27.4.12	25	59,0	-0,9	674	59	78 %	Const. press.
115244	Denmark	Skjaerne	56,0	8,1	82	3,1	-	-	2.4.12	21.6.12	80	72,0	-4,2	1874	156	32 %	Died and sank
115245	Denmark	Skjaerne	56,0	8,1	81	3	-	-	2.4.12	27.4.12	25	59,4	1,6	595	70	63 %	Const. press.
115247	Denmark	Skjaerne	56,0	8,1	86	4	-	-	2.4.12	27.4.12	25	59,3	-2,2	754	75	84 %	Const. press.
127794	Denmark	Varde	55,5	8,3	85	4,01	-	-	2.4.13	2.4.13	0	55,5	8,3	-	-	0 %	Died and sank
127798	Denmark	Varde	55,5	8,3	82	3,3	-	-	2.4.13	30.9.13	181	75,8	-4,3	2264	468	58 %	Const. press.
127799	Denmark	Varde	55,5	8,3	82	3,45	-	-	2.4.13	15.6.13	75	68,6	2,6	1436	108	95 %	Const. press.
127801	Denmark	Varde	55,5	8,3	77	2,2	-	-	2.4.13	14.4.13	12	55,9	7,5	97	23	68 %	Died and sank
128000	Denmark	Varde	55,5	8,3	94	5,1	-	-	2.4.13	30.9.13	182	71,5	-0,2	1786	258	75 %	Const. press.
128001	Denmark	Varde	55,5	8,3	85	3,6	-	-	2.4.13	24.5.13	52	55,7	8,7		11	4 %	Const. press.
128002	Denmark	Varde	55,5	8,3	84	3,9	-	-	2.4.13	2.4.13	0	55,5	8,3		#N/A	100 %	Const. press.
136044	Denmark	Varde	55,5	8,3	90	4,5	-	-	4.4.14	3.5.14	29	57,8	3,0		22	86 %	Const. press.
136045	Denmark	Varde	55,5	8,3	85,5	3,72	-	-	4.4.14	20.4.14	16	55,5	8,4	237	14	22 %	Predation
136047	Denmark	Varde	55,5	8,3	80	3,06	-	-	4.4.14	11.4.14	7	56,9	5,9	153	57	80 %	Const. press.
136048	Denmark	Varde	55,5	8,3	91	4,518	-	-	4.4.14	16.8.14	134	72,3	-0,3	1922	231		Died and sank
136051	Denmark	Varde	55,5	8,3	76	2,692	-	-	4.4.14	31.8.14	149	71,5	-13,0	2018	231	54 %	Died and sank
136052	Denmark	Varde	55,5	8,3	76	2,4	-	-	4.4.14	12.6.14	69	62,9	-18,6	1554	248	84 %	Predation
136053	Denmark	Varde	55,5	8,3	75	2,17	-	-	4.4.14	14.4.14	10	55,6	7,3	96	22	69 %	Const. press.
85520	Greenland	Nuuk	63,9	-51,4	69,5	4,3	3	1	13.9.10	4.4.11	203	56,8	-54,8	812	768	52 %	On time
85522	Greenland	Nuuk	63,9	-51,5	64	3,2	5	1	13.9.10	27.9.10	14	64,0	-53,1	80	209	99 %	Const. press.
85523	Greenland	Nuuk	63,9	-51,5	66,5	4,1	3	1	13.9.10	1.10.10	18	62,7	-50,1	149	70	91 %	Const. press.
85521	Greenland	Nuuk	63,9	-51,6	65,5	3,4	2	1	15.9.10	3.10.10	18	63,2	-45,9	293	231	100 %	Const. press.
107094	Greenland	Nuuk	63,9	-51,5	65	3,2	3	1	14.9.11	16.9.11	2	63,0	-52,3	111	92	100 %	Const. press.
107095	Greenland	Nuuk	63,9	-51,5	70,5	3,8	-	1	14.9.11	6.10.11	22	63,8	-52,9	130	48	100 %	Const. press.
107097	Greenland	Nuuk	64,0	-51,6	64	3,6	2	1	15.9.11	3.10.11	18	63,7	-52,8	68	307	100 %	Const. press.
107093	Greenland	Nuuk	64,0	-51,6	67	3,7	2	1	23.9.11	18.10.11	25	63,1	-51,5	158	145	100 %	Const. press.
107098	Greenland	Nuuk	64,0	-51,6	63,5	3,2	-	1	23.9.11	3.10.11	10	62,1	-56,1	314	38	100 %	Const. press.
107099	Greenland	Nuuk	64,0	-51,6	69	3,8	4	2	27.9.11	17.10.11	20	63,6	-52,0	48	38	96 %	Const. press.
107100	Greenland	Nuuk	64,0	-51,6	68	3,8	-	1	27.9.11	1.4.12	187	53,4	-43,2	1267	672	41 %	On time
107107	Greenland	Nuuk	64,0	-51,6	66,5	3,5	3	1	27.9.11	4.10.11	7	62,0	-55,7	303	75	100 %	Const. press.
107108	Greenland	Nuuk	64,0	-51,6	62,5	2,9	4	1	27.9.11	18.10.11	21	64,0	-52,9	151	54	98 %	Const. press.
34438	Iceland	Laxa	66,1	-17,6	80,7	3,3	2	2	4.5.11	2.6.11	29	63,5	-28,9	614	505	100 %	Const. press.
52526	Iceland	Laxa	66,1	-17,6	76,5	2,6	3	2	4.5.11	13.7.11	70	61,7	-37,6	1114	322	89 %	Const. press.
34436	Ireland	Blackw./Suir	52,1	-7,8	65,5	2,2	2	1	11.3.10	15.3.10	4	51,5	-6,6	110	86	91 %	Const. press.
34868	Ireland	Blackw./Suir	52,1	-7,8	78	3,1	2	2	11.3.10	15.3.10	4	51,6	-6,4	114	91	100 %	Recovered
34870	Ireland	Blackw./Suir	52,1	-7,8	76,5	3,3	2	2	11.3.10	20.3.10	9	51,3	-9,1	129	70	100 %	Recovered
34881	Ireland	Blackw./Suir	52,1	-7,8	69	2,3	3	1	11.3.10	22.8.10	165	69,0	2,3	-	757	100 %	Recovered
34438	Ireland	Blackw./Suir	52,1	-7,8	74,5	2,9	2	2	11.3.10	12.3.10	1	51,9	-5,7	145	70	100 %	Recovered
34439	Ireland	Blackw./Suir	52,1	-7,8	70,5	2,8	2	1	11.3.10	1.7.10	112	53,9	-43,9	2401	870	79 %	On time
34867	Ireland	Blackw./Suir	52,1	-7,8	66,5	2,3	3	1	25.3.10	4.4.10	10	51,2	-11,4	266	177	75 %	Const. press.
34877	Ireland	Blackw./Suir	52,1	-7,8	83,5	3,9	3	1	25.3.10	27.5.10	63	58,3	-26,5	1143	306	92 %	Predation
35086	Ireland	Blackw./Suir	52,1	-7,8	79	3,8	2	2	25.3.10	6.6.10	73	57,7	-33,8	1737	693	90 %	Predation
34866	Ireland	Blackw./Suir	52,1	-7,8	80,5	3,7	2	2	25.3.10	24.5.10	61	59,7	-23,3	1006	709	74 %	Const. press.
34870	Ireland	Suir/Nore/Barrow	52,1	-7,0	81,8	4	3	2	11.3.11	20.3.11	9	51,3	-9,5	197	64	100 %	Recovered
34440	Ireland	Suir/Nore/Barrow	52,1	-7,0	69,4	2,7	3	1	18.3.11	13.8.11	148	63,9	-28,3	1738	371	79 %	On time
34460	Ireland	Suir/Nore/Barrow	52,1	-7,0	68,6	2,3	2	1	18.3.11	1.6.11	75	58,5	-14,3	979	413	26 %	Predation
34472	Ireland	Suir/Nore/Barrow	52,1	-7,0	68,3	2,3	2	1	18.3.11	3.4.11	16	53,6	-11,2	350	102	97 %	Const. press.
34868	Ireland	Suir/Nore/Barrow	52,1	-7,0	72,5	3	2	2	18.3.11	5.4.11	18	51,7	-6,4	60	91	100 %	Recovered
34439	Ireland	Suir/Nore/Barrow	52,1	-7,0	63,2	2,1	2	1	18.3.11	6.5.11	49	54,1	-14,0	518	381	100 %	Recovered
34866	Ireland	Suir/Nore/Barrow	52,1	-7,0	75,5	3,4	3	2	18.3.11	9.5.11	52	56,5	-15,0	686	258	82 %	Predation
34867	Ireland	Suir/Nore/Barrow	52,1	-7,0	71,2	2,7	3	2	18.3.11	25.8.11	160	62,5	-33,6	1947	639	84 %	Const. press.
34877	Ireland	Suir/Nore/Barrow	52,1	-7,0	71	2,8	2	2	18.3.11	6.4.11	19	51,9	-7,3	32	75	89 %	Const. press.
127791	Spain	Lerez	42,4	-8,8	73	2,2	-	-	14.3.13	22.3.13	8	43,6	-9,6	153	64	100 %	Died and sank
127792	Spain	Lerez	42,4	-8,8	77	3,1	-	-	14.3.13	1.4.13	18	53,7	-17,0	1398	32	96 %	Predation
127793	Spain	Lerez	42,4	-8,8	78	2,9	-	-	14.3.13	7.4.13	25	49,5	-15,9	961	118	85 %	Predation
127795	Spain	Lerez	42,4	-8,8	74	2,8	-	-	14.3.13	27.5.14	439	62,1	-39,2	2965	560	91 %	Const.bpress.
127797	Spain	Lerez	42,4	-8,8	73	2,4	-	-	14.3.13	6.5.13	53	56,2	-12,0	1555	124	75 %	Const. press.
128004	Spain	Lerez	42,4	-8,8	72	2,3	-	-	14.3.13	21.3.13	7	47,3	-6,3	578	32	72 %	Predation
136037	Spain	Lerez	42,4	-8,8	91,5	4,6	-	-	18.3.14	25.3.14	8	42,0	-21,6	1061	301	97 %	Const. press.
136038	Spain	Lerez	42,4	-8,8	84	4,2	-	-	18.3.14	25.3.14	7	45,2	10,6	1578	22	91 %	Predation
136040	Spain	Lerez	42,4	-8,8	78	3,5	-	-	18.3.14	12.4.14	26	42,5	-9,6	121	420	75 %	Const. press.
136041	Spain	Lerez	42,4	-8,8	82,5	3,2	-	-	18.3.14	12.4.14	25	55,8	-12,1	1505	16	97 %	Const. press.
136043	Spain	Lerez	42,4	-8,8	80	2,3	-	-	18.3.14	12.4.14	25	43,1	-12,6	326	355	97 %	Const. press.
